# Artificial Intelligence in Interventional Pain Management: Opportunities, Challenges, and Future Directions

**DOI:** 10.37825/2239-9747.1064

**Published:** 2024-11-21

**Authors:** Matteo Luigi Giuseppe Leoni, Marco Mercieri, Giustino Varrassi, Marco Cascella

**Affiliations:** a Department of Medical and Surgical Sciences and Translational Medicine, Sapienza University of Rome, Rome, Italy; b Fondazione Paolo Procacci, 00193, Rome, Italy; c Department of Medicine, Surgery and Dentistry, University of Salerno, 84081, Baronissi, Italy

*Dear Editor*,

Artificial intelligence (AI) and machine learning have already made significant advancements in medicine and healthcare [1]. In fact, these technologies have the potential to revolutionize how healthcare professionals diagnose, treat, and monitor patients. By leveraging vast amounts of digitalized medical data, AI can enhance diagnostic accuracy, enabling more precise and personalized care. This integration of AI into healthcare not only bridges the gap between data and clinical decisions but also optimizes healthcare delivery, paving the way for more efficient and effective patient care. With AI’s ability to analyse and learn from complex datasets, it can support healthcare providers in making informed decisions, predicting patient outcomes, and tailoring treatments to individual needs, ultimately improving overall healthcare quality and patient outcomes. Probably, the term “Augmented Intelligence” might be more appropriate than “Artificial Intelligence” because it highlights AI’s function as a partner that enhances human intelligence rather than substituting it. However, establishing clear guidelines and regulations for augmented intelligence is crucial for successfully incorporating AI into healthcare teams.

This commentary discusses the critical considerations for integrating AI into interventional pain management.

## 1. Actual and future applications for AI in interventional pain management

AI application in interventional pain management, particularly for spinal pain, is gaining significant attention. The integration of AI in this domain promises to enhance precision, improve patient outcomes, and optimize clinical workflows.

Here are some examples of AI applications in interventional pain management, along with potential advancements:

1. *Interpretation of diagnostic images*. The use of AI in interventional pain management required the frequent integration of advanced imaging technologies such as MRI and CT. AI systems can effectively process and analyse these images to provide accurate guidance during procedures. For example, convolutional neural networks have shown promising results in interpreting spinal imaging with high accuracy, leading to significant improvements in spinal surgery outcomes when guided by AI technology [2]. AI can also determine the most appropriate MRI protocol for each individual patient, reducing excessive scanning time or minimizing radiation exposure in CT examinations. Additionally, AI can significantly reduce Gibbs artifacts in imaging by applying advanced algorithms that detect and correct these distortions, thereby enhancing overall image quality [3].
2. *Optimization of interventional images*. AI has the potential to revolutionize image optimization in pain management procedures using fluoroscopy and ultrasound. By integrating AI technologies into these machines, they can analyse imaging patterns, such as dye spread in fluoroscopy or tissue characteristics in ultrasound, to enhance procedural precision. AI can also distinguish between optimal and suboptimal images, offering real-time recommendations to improve physician technique [4]. Through pattern recognition, AI can identify ideal zones for targeting and the best trajectory to reach a specific target, significantly increasing the accuracy and safety of procedures.
3. *Robotics and Precision AI-assisted robotic systems*. These systems can assist in performing surgery or minimally invasive techniques by enhancing intraoperative navigation, reducing the risk of complications, and minimizing iatrogenic injury. Although robotic-assisted surgery is still in the early stages of development, it holds the potential to improve surgeons’ technical precision and enhance patient outcomes.
4. *Holographic and Augmented Reality Displays*. Incorporating holographic or augmented reality displays into surgical planning can provide real-time visualization of a patient’s anatomy, including anatomical variations, thereby enabling personalized management and tailored surgical strategies. These technologies can enhance the surgeon’s ability to make informed decisions and precisely target specific areas [5].
5. *Big data management and outcome prediction*. Integrating patients’ data with electronic health records into AI systems can provide comprehensive patient histories, current health status, and predictive analytics to guide treatment decisions effectively. Predictive models, powered by advanced AI and machine learning algorithms, can detect patterns and predict outcomes in interventional pain management, leading to better patient outcomes [6]. By analysing extensive data, including medical histories, imaging results, and biometric information, these models can identify hidden patterns and risk factors, allowing for the precise selection of the most appropriate interventional procedures [7].
6. *Training for Minimally Invasive Pain Management*. AI has the potential to revolutionize the training of young residents in minimally invasive pain management procedures. By creating realistic virtual environments, AI allows trainees to practice techniques repeatedly, developing essential motor skills with real-time feedback [8]. This personalized approach accelerates learning and builds confidence, ensuring proficiency before real-life application. Additionally, AI can analyse data from past surgeries to enhance decision-making and standardize best practices, making it a powerful tool in the education of future physicians.

## 2. Challenges and considerations for integrating AI in interventional pain management

However, despite the various potential advantages of AI technologies, not all issues have been fully resolved [9]. In fact, before integrating AI into interventional pain management, several potential issues and areas for improvement must be addressed:

1. *Quality of data*. One major concern is the quality of data. As AI systems rely heavily on large datasets that need to be accurate, consistent, and representative of diverse patient populations. Inadequate or biased data can lead to erroneous conclusions and compromised patient care. A significant challenge is the difficulty in collecting large amounts of high-quality data, which is crucial for training effective AI models. In fact, combining data from different pain centers is often challenging due to difficulties in creating specific registries, the absence of standardized platforms for data collection, and variability in clinical protocols across institutions.
2. *Cybersecurity*. Protecting patient data and data privacy is of paramount importance. Cyberattacks can have severe consequences on patient care, making it imperative to adopt stringent security protocols. Robust cybersecurity measures are essential to safeguard sensitive medical information and ensure the integrity and confidentiality of patient records.
3. *Regulatory Considerations*. Regulatory approval of AI technologies used in interventional pain management must comply with regulatory standards and obtain necessary approvals from the Food and Drug Administration (FDA) and European Medicines Agency (EMA). Ensuring that these technologies meet safety and efficacy standards is crucial. In fact, the FDA has outlined a framework for the regulation of AI in healthcare, emphasizing the need for transparency and validation [10].
4. *Ethical Considerations*. Ethical use of AI includes ensuring transparency in AI decision-making processes and maintaining human oversight to prevent over-reliance on automated systems. Ethical frameworks must be developed to address issues such as bias, accountability, and patient consent.
5. *Rules, guidelines and regulations*. There is a need for robust guidelines and regulations to govern the use of AI in clinical settings, ensuring that these technologies are safely and effectively integrated into existing workflows.
6. *Clinical Training*. AI has the potential to automate routine tasks, allowing clinicians to focus on more complex aspects of patient care. However, proper training for adopting AI into clinical practice is crucial. Physicians must be adequately trained to use AI systems effectively, which includes understanding how to interpret AI-generated outputs and integrating these insights into their decision-making processes. This training ensures that clinicians can leverage AI technologies to enhance patient care while maintaining the critical human element in clinical interactions.
7. *Cost and Accessibility*. While the initial investment in AI technologies can be significant, conducting a thorough cost-benefit analysis is essential to evaluate the potential long-term savings from reduced complication rates, shorter hospital stays, and improved patient outcomes. Moreover, it is also crucial to address issues of accessibility. Efforts should be made to ensure that advancements in AI are accessible to a wide range of healthcare providers, including those in resource-limited settings, to ensure equitable benefits across the healthcare spectrum.

## 3. Future research and development

Ongoing research is essential for the continuous improvement of AI algorithms and robotic systems in healthcare. Collaboration among medical professionals, AI developers, engineers, and researchers is crucial to fostering innovation. Conducting rigorous clinical trials is vital to improve the evidence on the efficacy and safety of AI-assisted interventional pain management. In fact, evidence-based practice will be useful in achieving broader acceptance and implementation of these technologies.

**Fig. 1:**
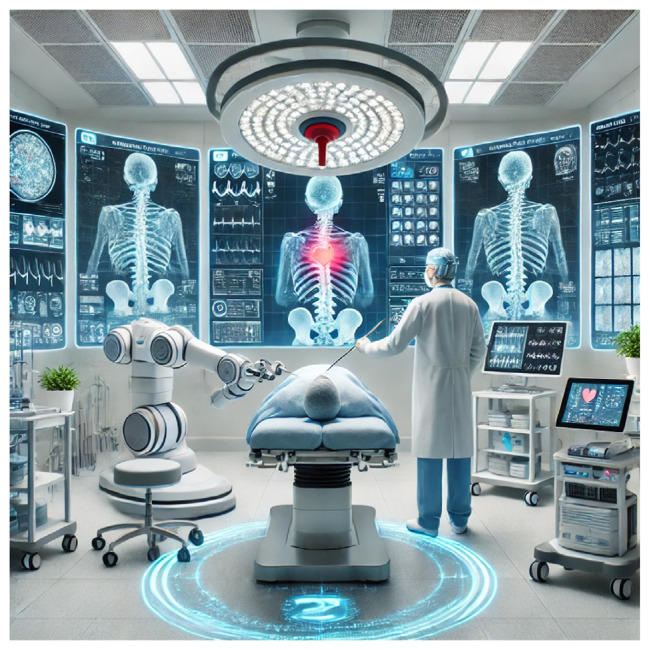
DALL-E interpretation of the viewpoint. DALLE Open AI 2024.

Finally, while integrating AI into interventional pain management presents numerous opportunities to enhance precision, improve patient outcomes, and optimize clinical workflows (Fig. 1), it is crucial to carefully consider the technological, ethical, regulatory, and practical aspects to ensure successful implementation and widespread adoption. Only by carefully addressing these factors we can fully leverage AI to transform pain management and enhance care for patients with chronic pain.
